# The ability of ‘non-cognitive’ traits to predict undergraduate performance in medical schools: a national linkage study

**DOI:** 10.1186/s12909-018-1201-7

**Published:** 2018-05-03

**Authors:** Gabrielle M. Finn, Lazaro Mwandigha, Lewis W. Paton, Paul A. Tiffin

**Affiliations:** 1 Hull York Medical School, University of York, Heslington, York, North Yorkshire YO10 5DD UK; 2 Health Sciences, University of York, York, UK

**Keywords:** UKCAT, Selection, Medical students, Personality, Conscientiousness, Undergraduate

## Abstract

**Background:**

In addition to the evaluation of educational attainment and intellectual ability there has been interest in the potential to select medical school applicants on non-academic qualities. Consequently, a battery of self-report measures concerned with assessing ‘non-cognitive’ traits was piloted as part of the UK Clinical Aptitude Test (UKCAT) administration to evaluate their potential to be used in selection.

**Methods:**

The four non-cognitive instruments piloted were: 1) the Libertarian-communitarian scale, (2) The NACE (narcissism, aloofness, confidence and empathy, (3) the MEARS (Managing emotions and resilience scale; self-esteem, optimism, control, self-discipline, emotional-nondefensiveness and faking, and (4) an abridged version of instruments (1) and (2) combined. Non-cognitive scores and sociodemographic characteristics were available for 14,387 applicants. A series of univariable and multivariable analyses were conducted in order to assess the ability of the non-cognitive scores to predict knowledge and skills-based performance, as well as the odds of passing each academic year at first attempt. Non-cognitive scores and medical performance were standardised within cohorts.

**Results:**

The scores on the non-cognitive scales showed only very small (magnitude of standardised betas< 0.2), though sometimes statistically significant (*p* < 0.01) univariable associations with subsequent performance on knowledge or skills-based assessments. The only statistically significant association between the non-cognitive scores and the probability of passing an academic year at first attempt was the narcissism score from one the abridged tests (OR 0.84,95% confidence intervals 0.71 to 0.97, *p* = 0.02).

**Conclusions:**

Our findings are consistent with previously published research. The tests had a very limited ability to predict undergraduate academic performance, though further research on identifying narcissism in medical students may be warranted. However, the validity of such self-report tools in high-stakes settings may be affected, making such instruments unlikely to add value within the selection process.

## Background

Entry to medicine is highly competitive. Applicants must excel academically, as well as achieving high scores in admissions tests and selection interviews. Medical schools must select from what may appear to be a homogenous cohort of applicants, in terms of applicants’ academic performance. It is for this reason that selection tests, such as the UK Clinical Aptitude Test (UKCAT) are useful, offering another mechanism by which to filter applicants.

The UKCAT is an admissions test taken by the vast majority of applicants to UK medical schools who are members of the UKCAT consortium. Originally the UKCAT consisted of four subscales that evaluated cognitive ability. These four cognitive sections evaluate abstract reasoning, verbal reasoning, decision making and quantitative reasoning. More recently a Situational Judgment Test (SJT) element was added [1, 2].

The scores on the cognitive sections of the UKCAT have been shown to have a modest, though statistically significant predictive validity, even after controlling for secondary (high) school attainment [3, 4]. Nevertheless, the constructs tested by such cognitive ability assessments will inevitably overlap with those measured by conventional educational attainment. Thus, there has been interest in evaluating non-academic qualities in order to add value within the selection process [5]. Desirable personal characteristics, such as empathy, may enhance the ability of the student to participate in educational activities, especially group tasks, such as study groups. This may lead to enhanced academic performance [6]. Likewise, individuals with high levels of less positive traits, such as narcissism, may have poorer educational performance [6]. Self-report personality traits have been shown to demonstrate some correlation with subsequent performance in medical school [7]. However, it is not clear which personal qualities may be most associated with positive academic undergraduate achievement.

Historically, in medical selection, evidence in relation to personality traits has been sought via personal statements and references as well as the use of unstructured face-to-face interviews; methods that now considered to have poor predictive validity [8, 9]. Attention has thus shifted to other means of evaluating such traits, such as Situational Judgment Tests [10], Multiple-Mini Interviews [11]. Personality tests have some attractive features in that they are cheap and efficient to mass administer, score and interpret. Many such tests also have established reliability and validity in a number of settings, if not in the context of medical selection. However, there have been relative few studies examining the relationship between such test scores and subsequent educational performance. Those that have been published tend to be single site evaluations [12–15].

For these reasons a battery of self-report personal qualities instruments were piloted as part of the UKCAT tests procedures. The UKCAT piloted such questionnaires between 2007 and 2010. For convenience we refer to these instruments as the ‘non-cognitive’ tests, though accept even such non-academic qualities will have cognitive components to them (e.g. ‘situational cognition’). They were included exclusively as tests for research purposes. A previous study analysing these scores reported only very modest correlations between the non-cognitive traits and Educational Performance Decile (EPM) in medical entrants [5]. It was noted, however, that emotional non-defensiveness was an independent and statistically significant predictor of EPM [5]. However, EPM is a somewhat crude measure that attempts to summarise the relative performance of a student throughout 5 years of medical undergraduate study [5]. In undertaking more detailed analyses it was hoped that more subtle patterns of association between the different aspects of undergraduate performance and personal qualities could be elicited. In particular we hypothesised that non-cognitive traits may have a specific impact on skills-based assessments, which are more likely to have an interpersonal element to them, compared to knowledge-based examinations. Thus, the purpose of this study was to establish whether non-cognitive assessments used within the UKCAT examination had any predictive validity for performance at medical school.

## Methods

### Ethics

The study was a secondary analysis of de-identified data. The participating UKCAT candidates were informed that their data would be used for research purposes. Thus, the study did not require an ethical opinion or approval. This was confirmed in writing by Durham University’s School for Health Ethics Committee.

### Data preparation

Data were collated for 14, 387 applicants who completed the UKCAT cognitive tests between 2007 and 2010. Data were provided by the University of Dundee Health Informatics Centre (HIC) on behalf of UKCAT. Data were matched for each applicant; these data included demographic data from applicants’ UKCAT registration and Universities and Colleges Admissions Service (UCAS) application. Medical school performance outcomes (obtained from the consortium medical schools by UKCAT) were also linked for each applicant where available.

### Predictor variables

For the present study, the UKCAT scores and year of sitting were available. Applicants wishing to enter a UKCAT consortium medical school must sit the test the preceding year (e.g. from July to October 2017, for entry in October 2018). There is not a limit on the number of times the test may be taken, although it can only be sat once for each admission cycle. In this study, the four scale scores and total scores were standardised as z-scores within each cohort of test-takers (including unsuccessful applicants) for that year. In the case of those that had taken the UKCAT multiple times, the scores from the most recent sitting were used. This course of action was taken because these will have been the level of UKCAT performance used by the admitting universities to make the decision to offer a place.

Between 2007 and 2010, UKCAT piloted five additional tests, which aimed to measure personal qualities (which are frequently termed as ‘non-cognitive’). UKCAT recorded that the non-cognitive (section 5) was introduced in 2007, on a pilot trial basis (UKCAT 2008). Section 5 was designed to identify additional attributes and characteristics that contribute to success in either medicine or dentistry careers; *robustness*, *empathy* and *integrity* (UKCAT, 2007).

Tests were allocated to candidates randomly by Pearson VUE (see Table 1 for allocation distribution). All scales were piloted but the scores were never used within the selection processes at any medical schools. Applicants were aware that the scores from these pilot tests would not be utilised within selection.

**Table 1:** Non-cognitive test allocation data

Year	Tests Used	Candidate numbers
2007	*ITQ100*	6004
	*IVQ49*	5928
	*ITQ50/IVQ33*	5808
	*MEARS*	2445
2008	*ITQ100*	4557
	*IVQ49*	4397
	*ITQ50/IVQ33*	4661
	*MEARS*	6896
2009	*ITQ100*	4790
	*IVQ49*	4772
	*ITQ50/IVQ33*	4738
	*MEARS*	4701
	*SAI2*	4720
2010	*ITQ50/IVQ33*	8338
	*MEARS*	8400
	*SAI2*	8406

The non-cognitive section of the UKCAT included five tests over this time period;

- The Managing Emotions and Resilience Scales (MEARS) – comprised of the domains *self-esteem*, *optimism*, *self-discipline*, *faking*, *emotional non-defensiveness*, and *control.*
- The Interpersonal Values Questionnaire (IVQ) or *libertarian-communitarian* domain (Libcom)- this purports to measure the extent to which the respondent favours individual freedoms (versus societal rules) as a basis for making moral decisions.
- The Interpersonal Traits Questionnaire (ITQ) or NACE – comprised of the domains *narcissism*, *apathy*, *confidence* and *empathy*. This estimates self-reported *narcissism*, *aloofness*, *confidence* (in dealing with people) and *empathy* and produces a summary score for *involvement* (versus *detachment*) in which *confidence* (C) and *empathy* (E) are positive; *narcissism* (N) and *aloofness* (A) are negative (the ‘NACE’ score).
- The Self-Appraisal Inventory (SAI), which measures the domains of (mental) *resilience* (comprising scales measuring *anxiety*, *moodiness*, *neuroticism* and *irrational thinking*) and *self-control* (versus risk taking tendency) using the scales of *restraint*, *conscientiousness*, *permissiveness* and *anti-social tendencies*. The SAI also contains a *lie scale*.
- Abridged versions of the IVQ and ITQ (ITQ50/IVQ33).

As with the cognitive scales, the non-cognitive scale scores were standardised within cohorts. Test scoring was conducted by Pearson VUE and the subscale summary scores were made available to the authors. The exception was the overall NACE summary score, generated by the research team ([*Confidence* + *Empathy*] – [*Narcissism* + *Aloofness*]).

Literature on the reliability and validity of the scales utilised by UKCAT is limited [5]. However, reliability coefficients ranging between 0.80 and 0.95 have been reported for some of the individual scales [16]. Some scales have demonstrated predictive validity for tutor ratings of personal qualities in year 1 and year 2 of undergraduate medicine [13]. These later studies provide some evidence for convergent validity for these scales [13, 15–17] . The present study aims to go some way towards providing evidence for the predictive validity of the scales in question (both convergent and divergent) in relation to undergraduate academic performance.

In addition to the UKCAT cognitive and non-cognitive section scores, data on demographic and prior academic achievement were available. These data were linked to the UKCAT dataset by the authors and are summarised in Table 2. As with our previous studies of the UKCAT, a continuous metric of academic performance that included Irish and Scottish qualifications as well as A-levels was created [8, 18]. This metric was derived by summarising the examination results as a percentage of the maximum achievable UCAS tariff scores that could be obtained. Standardised z scores were then derived within students for each nationality (i.e. authors compared all those taking Scottish “higher” qualifications against each other). The three highest grades were included but this excluded ‘General Studies’. Conforming to entry requirements for medicine, a significant proportion of subjects taken at A-level were science or mathematics. With respect to Scottish Highers and Irish leaving certificates (ILC), the best of five or six exams were utilised, respectively. As with the A-levels, Irish and Scottish exams were mostly taken in mathematics and the sciences, however significant numbers of students also took other subjects such as English, French and geography. In accordance with common entry requirement to Irish medical schools, Irish was also frequently studied for the ILC. In all cases, only the grades at first sitting were retained. Exclusions from the dataset included candidates who did not have the minimum number of advanced qualifications required (for example, fewer than three A-level passes). Consequently, management of the advanced educational qualification data emulated the approach typically used by UK medical schools in appraising predicted or achieved secondary school qualifications. The UKCAT database records reported socioeconomic status using a simplified version of the socioeconomic classification system used by the National Office for Statistics [19]. Approaches utilised in previous research in this area were adopted, thus authors classified those who gave themselves a socioeconomic classification rating of four or more as being from a ‘non-professional’ background, ethnicity was dichotomised into White and Non-white, and schools into selective (independent and grammar schools) and non-selective (state schools and sixth form colleges). In addition, age was dichotomised into those who were 21 or older at medical school entry (i.e. ‘mature students’) and those that were younger on admission, using the date of birth. Applicants who may have special educational needs (SENs) may also apply for SEN status for the purposes of sitting the UKCAT. This permits such candidates additional time to complete the test and the SEN status of applicants was included in the study dataset.

**Table 2:** Demographic data

Variable	Proportion (%)	Missing (%)
Male sex	6310 (43.9)	0 (0)
Age ≥ 21 years at entry	2699 (19.0)	202 (1.4)
Non-selective school attended	5556 (46.9)	2540 (17.7)
Non-white ethnicity	4038 (29.0)	448 (3.1)
Non-professional socioeconomic background	470 (3.7)	1586 (11.0)
Registered as special educational needs for UKCAT	320 (2.2)	0 (0)
English as a second language	1549 (22.2)	7395 (51.4)

### Outcome variables

In each year at medical school, typically five in the United Kingdom, students sit a range of examination types. These data were collated and categorised as ‘knowledge’ or ‘skills’ prior to the dataset being released to the authors. The overall outcome for each year at medical school (e.g. pass year at first attempt, and so on) was also available. The raw knowledge and skills scores were given as percentages, which were local scores for each year and specific medical school. Therefore, they were transformed into standardised z scores (mean zero and SD of 1) for each year and medical school, to allow for some comparison. Thus, the z-score for medical school performance was the dependent variable in this context.

The end of year results (e.g. pass at resit), for the purposes of analysis, were dichotomised into ‘*pass first time*’ versus any other academic outcome (e.g. *pass after resit or resit the year*). Different universities may have had differing standards for passing the year. Therefore, the outcome for each end of year exam had to be considered hierarchical in nature because the outcomes were nested within universities. The odds of passing *any* year first time were modelled with the students (nested within universities) considered a unit of clustering (i.e. in effect, this is analogous to a three level multilevel model being used). The universities and students can thus be potentially considered to be clusters. Therefore, models which could handle, and correct for, the clustered nature of the data were used.

### Data analysis

The association between subscale scores and sociodemographic variables were explored using linear regression. For the continuous *knowledge* and *skills* outcomes, linear mixed models (LMM) were adopted to assess the association between the outcomes with subscale scores, allowing for a clustering effect for universities (by introducing a random intercept into the model). In this case the model was, in effect, a two-level model, as only one exam outcome per year per student was evaluated. Thus, there were no student-level clustering effects as such in contrast to what would be the case if the overall end of year outcomes for all years were analysed simultaneously for each student [20].

For the dichotomous outcome *pass first time,* a Generalised Estimating Equations (GEE) framework was used to assess the association between *pass first time* and the subscales of interest. Both the LMM and GEE handle clustered data appropriately by correcting for the downward bias in standard errors introduced by the fact that outcomes *within* a university are more likely to be similar than those *between* institutions [21].

In the case of the dichotomous outcome *pass first time*, GEE was used instead of GLMM for several reasons. In contrast to GEE models, GLMM models using a non-linear link function (e.g. probit or logit) do not have a marginal interpretation (that is, a population average interpretation), hence GEE was preferred. On the other hand, if the outcome of a multi-level model is continuous (as in our case ‘knowledge’ and ‘skills’ based exams), from the multi-level model a marginal interpretation (that is population average interpretation) may be made. That is why the continuous outcomes were modelled using a GLMM (multi-level) approach [21].

Data exploration and manipulation was conducted using R statistical software [19] while the statistical modelling for this paper was generated using SAS version 9.4 [22]. The importance of associations was evaluated at an 5% a priori alpha level without any corrections for multiple comparison. Missing values were treated via listwise deletion.

## Results

### Non-cognitive scores as predictors of academic performance in medical school

The results for each non-cognitive assessment are presented below. The figures presented depict the standardised regression coefficients and associated 95% confidence intervals for predicting undergraduate performance, across corresponding outcomes by years and non-cognitive subscales. The coefficients were standardised according to the applicant scores as well as the medical school outcomes. Therefore the coefficients could be interpreted as the number of standard deviations (SDs), above the mean scored for medical students in a particular cohort and university for every SD scored, above the mean for the applicants who took that particular scale. The effect sizes were generally less than 0.2. In this instance, an effect size of 0.2 would be interpreted as follows; for every SD scored above the mean for applicants taking a particular non-cognitive scale a testee would, on average, achieve a medical school assessment score of 0.2 SDs above the mean for their specific cohort and university. Also, overall, as applicants progressed through their respective medical schools, the ability of the non-cognitive test scores to predict undergraduate academic performance waned.

**Fig. 1:**
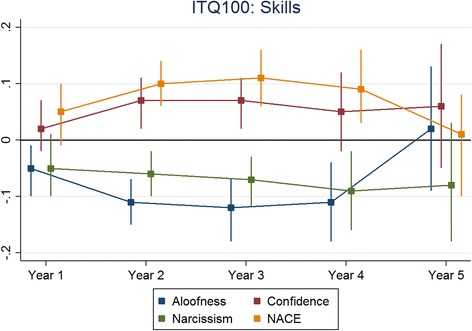
The predictive validity of ITQ100 for skills scores

**Fig. 2:**
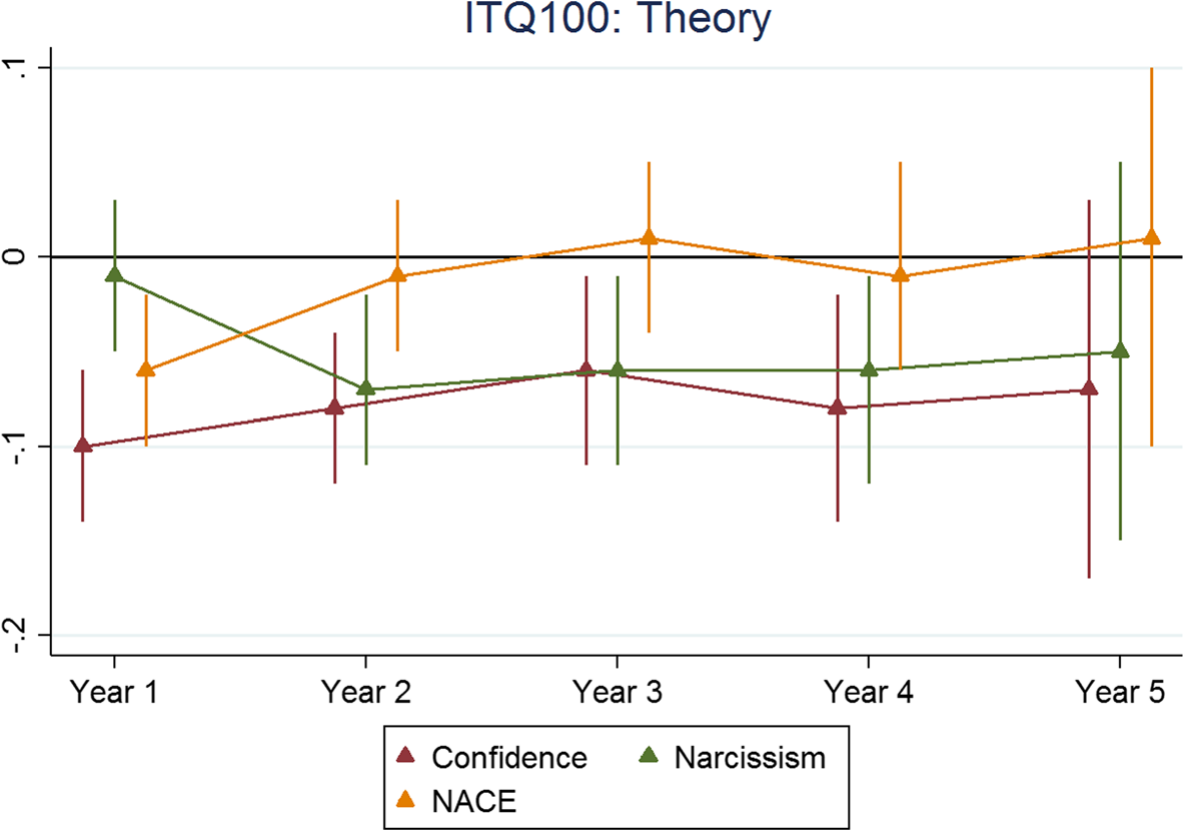
The predictive validity of ITQ100 for theory (knowledge) scores

**Fig. 3:**
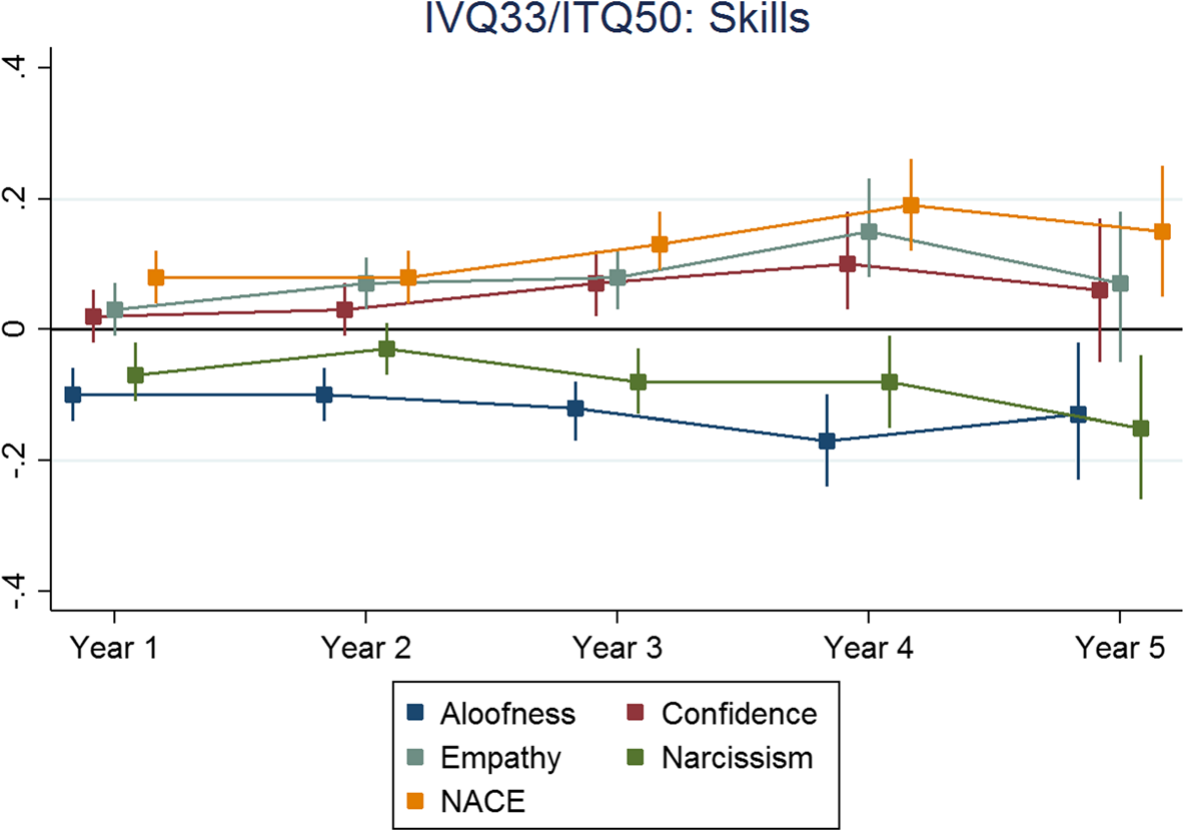
The predictive validity of IVQ33/ITQ50 for skills scores

**Fig. 4:**
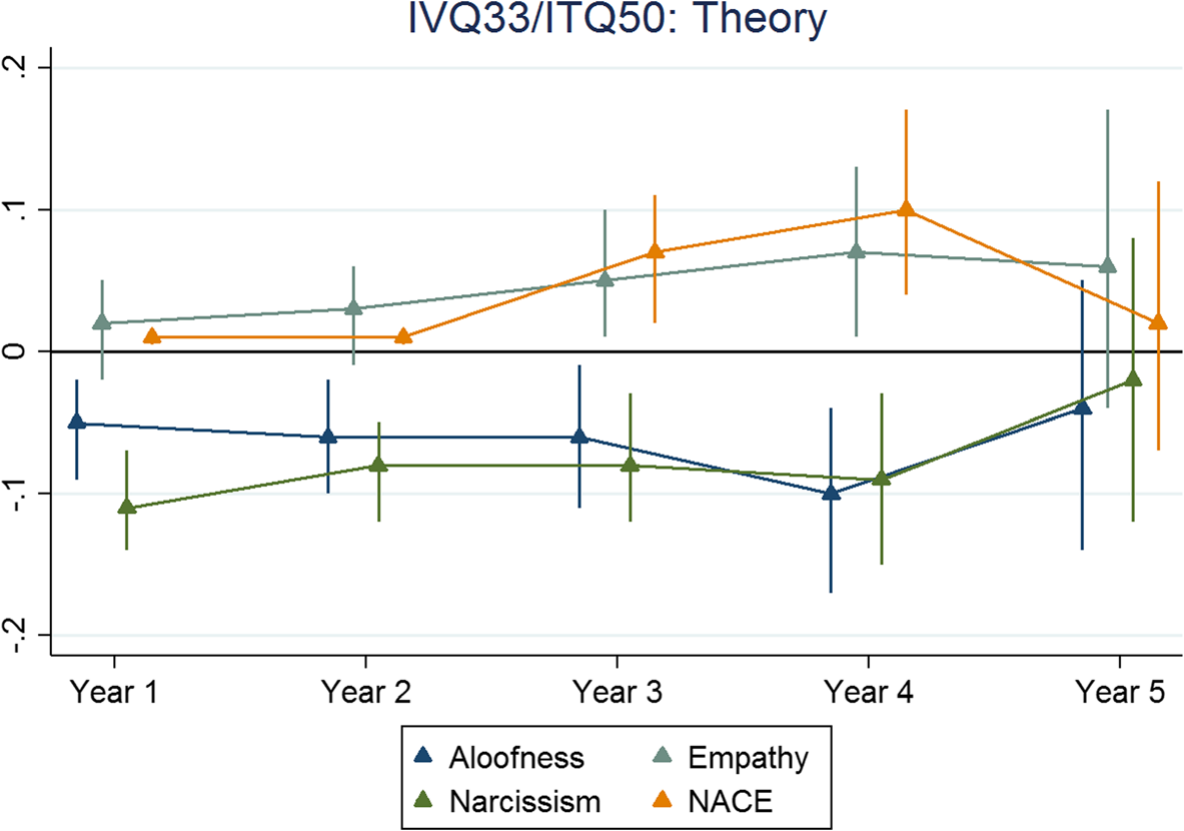
The predictive validity of IVQ33/ITQ50 for theory (knowledge) scores

**Fig. 5:**
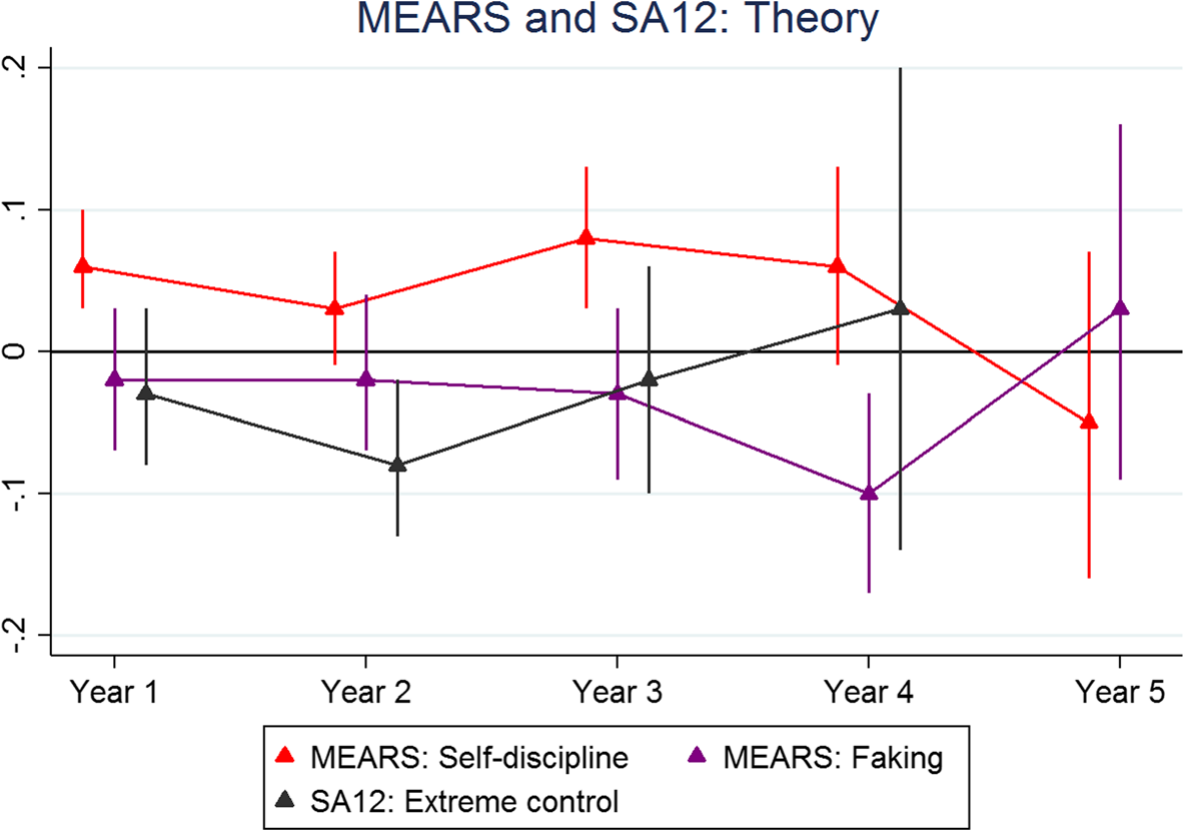
The predictive validity of MEARS and SAI2 for theory (knowledge) scores

**Fig. 6:**
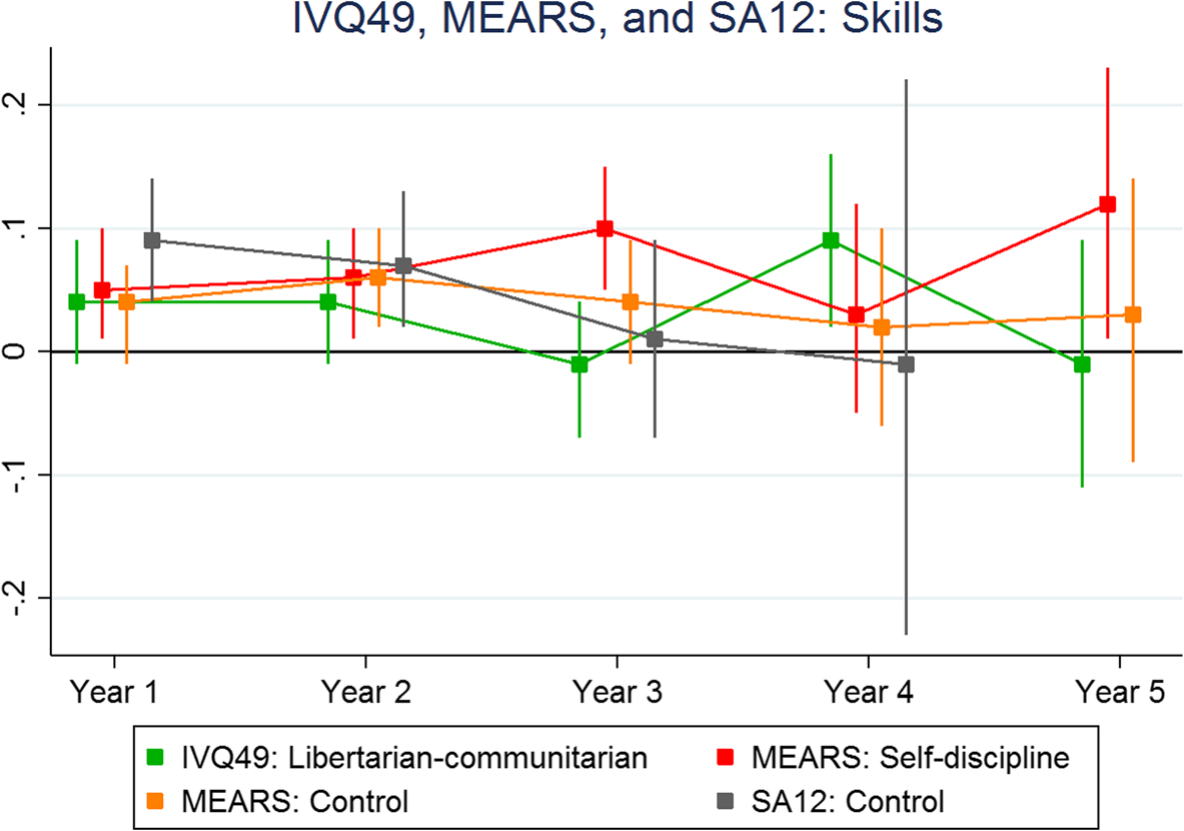
The predictive validity of IVQ49, MEARS and SAI2 for skills scores

Figure 1 shows the results for the ITQ100 predicting skills and Fig. 2 shows the corresponding results for knowledge-based outcomes. Figures 3 and 4 depict the results for the IVQ33/ITQ50 for skills and knowledge, respectively. Figure 5 demonstrate the corresponding results for the MEARS and SAI2 tests and knowledge. Similarly, Fig. 6 represent the results for MEARS and IVQ49 with skills-based academic outcomes.

### The ability of the UKCAT non-cognitive scores to predict passing a year at first attempt (i.E. a favourable academic outcome)

The results below use the (standardised) non-cognitive test scores to predict a favourable academic outcome (whether a student passed at first attempt or had another academic outcome). Table 3 uses the non-cognitive (standardised) scores with and without a time variable (representing the number of years spent at medical school) entered into the model. In effect this weights the outcomes by year, as the later on in medical school the student is, the less likely they are to receive an unfavourable academic outcome. This may be partly due to poorer students having been eliminated earlier. However, these unsuccessful students are relatively few in the number in the UK. Thus it may also largely reflect a reluctance to fail students nearer the end of undergraduate training having invested so much in their education.

**Table 3:** Significant results from the GEE analysis used to assess the ability of the UKCAT non-cognitive scores to predict passing each year of medical school at the first attempt

Test	Subscale	Time considered?	OR	*p*-value	95% CI
IVQ33/ ITQ50	narcissism	No	0.83	0.019	(0.71, 0.97)
	narcissism	Yes	0.84	0.021	(0.71, 0.97)
IVQ33/ ITQ50	NACE	No	1.11	0.056	(1.00, 1.25)
	NACE	Yes	1.23	0.062	(0.99, 1.25)

The vast majority of non-cognitive scale scores did not show a significant association with the odds of passing a year at first sitting (versus other academic outcome). Only one scale score statistically significantly predicted the odds of passing each year at first attempt (the narcissism scale of the ITQ50) (Table 3). The odds ratio associated with this scale score was 0.84 (0.71 to 0.97). This can be interpreted as follows; for every standard deviation above the mean (for that group of UKCAT candidates completing the scale) an applicant scored, their odds of passing that year of medical school reduced by roughly 16%. Adjusting for the specific year of sitting made no substantial difference. In addition, results of borderline statistical significance were observed for the NACE scale of the IVQ33. Increasing scores on this scale appeared modestly predictive of passing a year at first attempt, even after adjusting for specific year of sitting (OR 1.23, 0.99 to 1.25) (see Table 3).

## Discussion

The results for each test demonstrate that the assessments piloted had limited predictive validity for academic performance (standardized *skills* and *knowledge* scores) over the candidate’s time at medical school. Similarly, the tests had limited predictive capabilities for end of year academic outcome (pass at first attempt). The small effect sizes observed, typically of less than 0.2, have limited educational significance, despite achieving statistical significance. However, when medical schools must select from such large pools of students achieving similar academic criteria, the impact of small effect sizes should not be dismissed.

Despite this, it is worth commenting on a few notable findings. Firstly, some generally modest associations with demographic variable were noted. For example, males generally scored considerably lower on the *extreme control* scale of the SAI2. In contrast those registered as having SEN scored relatively highly on this scale, perhaps reflecting a determination to overcome a developmental disability (such as dyslexia). Older applicants were also more prone to expressing libertarian-communitarian views- perhaps reflecting a less self-orientated perspective that comes with maturity. We also observed a pattern relating to the *aloofness* and *narcissism* scales; scores tended to be higher in non-White and those speaking English as a second language. These two traits could be conceptualised as closely related, reflecting a rather egocentric world view and high self-esteem, with a tendency to disregard others. In this case they may also reflect cultural differences. The scores on these scales were also observed to have a modest association with poorer academic performance at medical school. Thus, further, more detailed modelling would be required to attempt to understand whether they represent unhelpful traits or are merely proxy markers for cultural differences in non-UK students who may find undergraduate studies more challenging.

Obviously the potential for disadvantaging (or advantaging) certain groups when introducing certain selection measures must be considered. However, a clear distinction should be made between test or *item bias* and *differential item functioning*; in the former case one group genuinely is lower on the trait being tested; in the latter case they may not be, but certain test items may be tapping into constructs other than the one being tested and therefore some groups (e.g. men) may respond differently to them. It may be that certain groups are higher on desirable traits than others. Reassuringly, the observed associations with the UKCAT cognitive scale scores were very small, suggesting the questionnaires were not evaluating intellectual ability.

### Comparison with previous findings

There has been relatively little research on the personal qualities of medical students and academic performance, although several papers relate to the properties of some of the instruments piloted in the present study. The Personal Qualities Assessment (PQA) was developed to support medical selection and was a forerunner of some of the non-cognitive scales used in the present study, containing a *libertarian-communitarian* scale, as well as a NACE scale (originally an ‘ECAN’ score was calculated). The authors of the PQA highlighted its relative insensitivity to sociodemographic factors, and thus the potential to widen access to medicine [23]. A follow-up study by the group observed virtually no association with performance at medical school. The study included 626 students. Fourth year rankings were available for 411 (66%) students and objective structured clinical examination (OSCE) rankings for 335 (54%) of those consenting. No significant correlations were detected between separate elements of the PQA assessment and student performance. However, an algorithm advocated by the authors of the PQA was used to define ‘non-extreme’ character types on the *involved-detached* and on the *libertarian-communitarian* moral orientation scales [15]. There was a trend of borderline significance (*p* = 0.05) for such students to be ranked higher in OSCEs (average of 7.5% or 25 out of 335, *p* = 0.049). The group also presented (unpublished) findings to the UKCAT Board that suggested the IVQ and ITQ were complementary and could be used to classify applicants into different groups with different interpersonal styles [24]. This presentation also reported modest correlations between some of the instrument scores, tutor ratings and undergraduate exam results [24].

Previous analysis using the UKCAT non-cognitive scales, carried out by a team from The University of Aberdeen, explored whether the scores were predictive of performance at the end of medical school (e.g. Educational Performance Measure (EPM) and UK Foundation Programme Situational Judgement Test (SJT) scores). A modest correlation between total EPM and each of the individual MEARS domains (*r* = 0.255 to 0.449) was noted. They also observed that the self-esteem score was significantly associated with EPM decile but the coefficients were, again, very small. *Aloofness* and *empathy* domains in the NACE test were observed to be negatively associated with both SJT score and EPM decile [5]. This is roughly in line with our own findings of very weak associations between the non-cognitive scores and academic performance.

### Possible interpretations

It may be that, especially for knowledge-based exams, that there is little a priori reason to expect a strong correlation between undergraduate performance and personal qualities. The authors of the PQA seemed to have intended that such selection measures would be used to predict more distal performance in doctors, perhaps which involved team working and patient contact. Moreover, defining and obtaining a clear validity criterion is very challenging; it may be that supervisor ratings and high-fidelity simulations (such as OSCEs) generally represent the best available at present. The former have the disadvantage in that supervisors and peers only seem to be able to accurately differentiate candidates at extremes (i.e. very high or low on a trait or ability).

We noted a slight tendency for those from non-professional backgrounds to score slightly higher on the control scale of the SAI2. This may reflect determined individuals who have had to work hard to achieve academically and overcome natural social disadvantage. The ITQ100 subscale scores of *aloofness*, *confidence* and *narcissism* were shown to be negative predictors of performance on both *knowledge* and *skills* assessment performance across years 1 to 4. These may represent traits that reflect undesirable personality structures that make it more difficult for some students to take advantage of the ‘social learning’ environment, whereby knowledge and skills are shared amongst peers. Students high on these traits may appear somewhat unsociable and arrogant to peers. It is also possible that a small number of students reporting to be high on these personal qualities may have undiagnosed autism spectrum disorders (ASDs). The presence of an ASD would also make learning more difficult in other ways (for example, difficulties in contextualising and generalizing learning and problems undertaking group tasks).

Some modest negative prediction is observed for the NACE summary score and *knowledge* scores in year 1. However, those who score more highly on the NACE tend to do slightly better on the *skills* assessments in years 2 to 4. It may be that higher NACE scores reflect more pro-social individuals who perform better at inter-personal tasks, which may be included in *skills* assessments. We also note that the NACE scores derived from the IVQ33/ITQ50 had a relationship, albeit one of borderline statistical significance, with the odds of passing a year at medical school at first attempt; in contrast the NACE scores derived from the ITQ100 showed no relationship with this outcome. One explanation for this paradoxical observation may be that when the shortened version of the NACE was created, only the optimal items were retained (for example, those loading heavily on to the factors being measured); thus the shortened version is more predictive of outcomes.

### Strengths and limitations

A large number of UKCAT applicants took at least one piloted non-cognitive scale. The study could be said to be *overpowered*. That is, effect sizes that would be unlikely to be of educational significance may still be statistically significant, often at very small *p* values (e.g. < 0.001). Thus, whilst a number of statistically significant predictors of performance were observed, the effect sizes ranged from modest (i.e. < 0.3) to negligible (< 0.1).

At the time the test was introduced, non-cognitive assessments only took place at some medical schools and were limited to interviews. Thus, range was unlikely to be significantly directly restricted in entrants in relation to these traits. However, some indirect range restriction may have occurred if such qualities also impacted on the ability to perform well on selection measures such as high school attainment and the UKCAT cognitive tests. Nevertheless such attenuating affects may have had relatively little impact on the results as the scores were standardised according to applicants rather than entrants [25].

This study was challenging as the exact method of piloting and scoring of the non-cognitive tests were not clear, and some considerable time had elapsed between administration and analysis. The fact that test were administered to different candidates meant that the relationship between the scale scores could not be explored. Importantly our modelling assumes a linear relationship between the traits linked to the non-cognitive questionnaire scores. In contrast, the authors of the PQA postulate that the component scales can be used to cluster and classify individuals [15], with non-extreme scorers representing one particular group. This suggests a ‘circumplex model’ underlies responses to the package of scales contained the PQA. In psychometrics evidence for such models are relatively rare (though sometimes postulated) yet in the case of interpersonal traits there may be some empirical support for such models [26]. However, in practice modelling such non-linear relationships may be challenging. Classifying individuals based on arbitrary cut-off scores on several dimensions will result in informational loss. One option may be to use latent class models to group individuals. It may also be fruitful to model scores based on the deviation from the mean scores (i.e. extremeness of scoring).

We noted relationships between scores and year 5 outcomes tend to be less often significant. This may have been an artefact of the attrition of students from the dataset at this stage, resulting in reduced power to show a difference.

The data within this study are within a restricted range due to the sample being solely from applicants who were successful in gaining a place at medical school and to those for whom progression data had been provided by the respective institutions. Disattenuation and restriction of range were not compensated for. Compensation frequently leads to higher estimates. Moreover, no correction for the level of significance for multiple tests was made. However, it is the magnitude of the effects that should be of most interest to selectors, rather than the probability that the association is unlikely to have occurred by chance. Thus, such corrections (e.g. Bonferroni) would have added little to interpretation of the results.

It is important to consider, more broadly, what the potential impact of missing data was on the results.

From previous research using related data we have some clear evidence for missingness mechanisms. Previously we have used multiple imputation as a form of sensitivity analysis to evaluate the likely extent of the impact of any missingness on our results [27]. We concluded that missing sociodemographic data in the UKCAT dataset was likely to be missing at random (MAR) rather than non-ignorable. This was because the results we obtained from multiply imputed datasets were largely the same as those estimated with non-imputed data, using maximum likelihood estimation. In terms of outcomes, the main reason that data were missing is that some medical schools failed to return data for particular years. This didn’t seem related to any particular individual characteristics, with the missingness being at the level of the medical school rather than the individual, and thus was likely to be missing completely at random (MCAR). Assuming that data was either missing at random (MAR), or missing completely at random (MCAR) then when using a generalised linear models, which used maximum likelihood as the estimation method, then unbiased parameter estimates are likely to be recovered from the modelling. Thus, the missing data would have a minimal impact on our results. In the case of the generalised estimating equation (GEE) model the issue is slightly different in relation to missing data. Unlike maximum likelihood estimation, GEE generally assumes data to be MCAR, rather than MAR. However, in the present case we employed a weighted form of GEE. This placed more weight on observations with more complete data. Therefore we expect the impact of any missingness to have been minimal.

Furthermore, as can be seen from our results, almost all the analyses showed the relationship between the predictors and the outcomes of interest to be extremely weak. It could be argued, as we have stated in the paper, that none of the effect sizes observed were ‘educationally significant’ (even if they were statistically significant). That is, the weakness of the association between the predictor and the outcome suggested that the predictor variable should not be used within medical selection for that purpose (i.e. to select students likely to have better academic achievement). Thus, even if the missing data were non-ignorable then the bias would have to be extremely profound to have influenced our results the extent that our conclusions were substantially changed, in this instance. Thus we believe the missing data did not pose a threat to the validity of our findings.

It could be argued that the association between personal qualities and academic performance is likely to be weak. A failure to observe such associations could be said to support ‘divergent validity’ of the selection measure, as previous educational attainment and cognitive ability (as evaluated via aptitude tests) already predict these elements of future performance. Rather a more appropriate outcome to validate such tests against may be related to future professionalism as aspects of behaviour, such as misconduct issues. Such outcomes are currently being explored as part of a separate, ongoing study.

The overall conclusion that can be drawn is that the non-cognitive (personal qualities assessments) do not sufficiently discriminate between candidates to warrant their inclusion within medical and dental selection processes. The findings of this study relate only to the United Kingdom.

### Implications for practice, policy and future research

The generally weak associations between the scores on these self-report measures and academic outcomes in medical school do not suggest great promise as selection tools, at least if undergraduate performance is a key consideration. Moreover, if used in high-stakes situations then faking and social desirability effects may be more marked, lowering the predictive validity further. Nevertheless, it would be wrong to dismiss the importance of evaluating non-academic traits in selection. The emerging evidence for the predictive validity of SJTs for future performance, especially in later clinical practice-based scenarios, suggest such traits are vital in determining future work based performance. It may be that SJTs, that are known to be less prone to faking effects compared to self-report personality type questionnaires, are more effective at tapping into such traits.

When looking at the role of non-academic qualities in medical selection there are also philosophical and moral challenges. There are no platonic ideal personality types that make the perfect doctor. Medical specialties often demand differing emphases on personal style and temperament and there is some evidence for modest difference in personality between doctors from different specialties [28]. However, all medical roles demand pro-social traits (required for interaction with patients, or at least colleagues in all specialisms) and values consistent with ethical practice. Thus, it may be best to focus on the testing of these via methods that are less prone to faking effects.

## Conclusion

The findings of this study are in line with previous findings in that the non-cognitive scores are weakly correlated with both demographic factors and subsequent academic performance (especially skills-based assessments). Virtually no relationships with the UKCAT cognitive scores were observed. Our findings suggest that self-report questionnaires may not be an effective method of evaluating traits that are advantageous to undergraduate study. In contrast, our current, ongoing, research on student fitness to practice declarations may highlight some associations between certain personal qualities and professionalism. Thus, we suggest future research on non-academic qualities focuses on later clinical practice and professional behaviour.

## Abbreviations

ASD: Autism Spectrum Disorders
ECAN: Empathy, Confidence, Aloofness and Narcissism
EPM: Educational Performance Measure
GEE: Generalised Estimating Equation
GLMM: General Linear Mixed Methods
ILC: Irish Leaving Certificate
ITQ: Interpersonal Traits Questionnaire
IVQ: Interpersonal Values Questionnaire
LMM: Linear Mixed Methods
MAR: Missing At Random
MCAR: Missing Completely At Random
MEARS: Managing emotions and resilience scale; self-esteem
NACE: Narcissism, Aloofness, Confidence and Empathy
OR: Odds Ratio
OSCE: Objective Structured Clinical Examination
PQA: Personal Qualities Assessment
SAI: Self-Appraisal Inventory
SD: Standard Deviation
SEN: Special Educational Needs
SJT: Situational Judgement Test
UK: United Kingdom
UKCAT: United Kingdom Clinical Aptitude Test

## References

[1] Lievens F (2016). Widening access in selection using situational judgement tests: evidence from the UKCAT. Med Educ.

[2] Husbands A (2014). Predictive validity of the UK clinical aptitude test in the final years of medical school: a prospective cohort study. BMC Med Educ.

[3] Tiffin PA (2016). Predictive validity of the UKCAT for medical school undergraduate performance: a national prospective cohort study. BMC Med.

[4] McManus I (2013). The UKCAT-12 study: educational attainment, aptitude test performance, demographic and socio-economic contextual factors as predictors of first year outcome in a collaborative study of twelve UK medical schools. BMC Med.

[5] MacKenzie R (2017). Do personality traits assessed on medical school admission predict exit performance? A UK-wide longitudinal cohort study. Adv Health Sci Educ.

[6] Ronningstam E. Identifying and understanding the narcissistic personality. Oxford: Oxford University Press; 2005.

[7] Lievens F (2002). Medical students’ personality characteristics and academic performance: a five-factor model perspective. Med Educ.

[8] Cleland J (2012). Identifying best practice in the selection of medical students.

[9] Patterson F (2016). How effective are selection methods in medical education? A systematic review. Med Educ.

[10] Cousans F (2017). Evaluating the complementary roles of an SJT and academic assessment for entry into clinical practice. Adv Health Sci Educ.

[11] Eva KW (2009). Predictive validity of the multiple mini-interview for selecting medical trainees. Med Educ.

[12] Adam J, Dowell J, Greatrix R (2011). Use of UKCAT scores in student selection by U.K. medical schools, 2006-2010. BMC Med Educ.

[13] Adam J (2012). Can personal qualities of medical students predict in-course examination success and professional behaviour? An exploratory prospective cohort study. BMC Med Educ.

[14] Adam J (2015). Predictors of professional behaviour and academic outcomes in a UK medical school: a longitudinal cohort study. Med Teach.

[15] Dowell J (2011). Predictive validity of the personal qualities assessment for selection of medical students in Scotland. Med Teach.

[16] Bore M (2005). Selection of medical students according to their moral orientation. Med Educ.

[17] Bore M, Munro D, Powis D (2009). A comprehensive model for the selection of medical students. Med Teach.

[18] Tiffin PA. Understanding the dimensionality and reliability of the cognitive scales of the UK clinical aptitude test (UKCAT): report for the UKCAT board. UK: UKCAT; 2013. Summary version. https://www.ukcat.ac.uk. Accessed 9 Sept 2016.

[19] Team, R.C. A language and environment for statistical computing, R foundation for statistical computing, vol. 2016; 2012.

[20] Verbeke G. Linear mixed models for longitudinal data, in Linear mixed models in practice. UK: Springer; 1997. p. 63–153.

[21] Molenberghs G, Verbeke G. Models for discrete longitudinal data. Springer; 2005.

[22] Inc., S.I (2015). SAS software.

[23] Lumsden MA (2005). Assessment of personal qualities in relation to admission to medical school. Med Educ.

[24] Powis D, Munro D, Bore M (2010). Measurement of non-cognitive personal qualities - IVQ, ITQ and SAI in UKCAT 2007–2010.

[25] Muthén B, Muthén L (2015). MPlus version 7.

[26] Gaines SO (1997). Evaluating the Circumplexity of interpersonal traits and the manifestation of interpersonal traits in interpersonal trust. J Pers Soc Psychol.

[27] Tiffin PA, Dowell JS, McLachlan JC (2012). Widening access to UK medical education for under-represented socioeconomic groups: modelling the impact of the UKCAT in the 2009 cohort. BMJ.

[28] Clack GB. Is personality related to doctors’ specialty choice and job satisfaction? The medical school. London: King's College London; 2002.

